# Physically Plausible Spectral Reconstruction †

**DOI:** 10.3390/s20216399

**Published:** 2020-11-09

**Authors:** Yi-Tun Lin, Graham D. Finlayson

**Affiliations:** School of Computing Sciences, University of East Anglia, Norwich NR4 7TJ, UK; G.Finlayson@uea.ac.uk

**Keywords:** spectral reconstruction, hyperspectral imaging, multispectral imaging

## Abstract

Spectral reconstruction algorithms recover spectra from RGB sensor responses. Recent methods—with the very best algorithms using deep learning—can already solve this problem with good spectral accuracy. However, the recovered spectra are physically incorrect in that they do not induce the RGBs from which they are recovered. Moreover, if the exposure of the RGB image changes then the recovery performance often degrades significantly—i.e., most contemporary methods only work for a fixed exposure. In this paper, we develop a physically accurate recovery method: the spectra we recover provably induce the same RGBs. Key to our approach is the idea that the set of spectra that integrate to the same RGB can be expressed as the sum of a unique fundamental metamer (spanned by the camera’s spectral sensitivities and linearly related to the RGB) and a linear combination of a vector space of metameric blacks (orthogonal to the spectral sensitivities). Physically plausible spectral recovery resorts to finding a spectrum that adheres to the fundamental metamer plus metameric black decomposition. To further ensure spectral recovery that is robust to changes in exposure, we incorporate exposure changes in the training stage of the developed method. In experiments we evaluate how well the methods recover spectra and predict the actual RGBs and RGBs under different viewing conditions (changing illuminations and/or cameras). The results show that our method generally improves the state-of-the-art spectral recovery (with more stabilized performance when exposure varies) and provides zero colorimetric error. Moreover, our method significantly improves the color fidelity under different viewing conditions, with up to a 60% reduction in some cases.

## 1. Introduction

Hyperspectral imaging devices are developed to capture scene radiance spectra at high spectral resolution. In the context of machine vision, hyperspectral imaging distinguishes different material properties at pixel level, which is commonly used in remote sensing [1,2,3,4,5], anomaly detection [6] and medical imaging [7,8]. Furthermore, the devices (sensors or displays), light sources and object surfaces are commonly characterized by spectral measurements [9,10,11]. Practical applications include scene relighting [12] and digital art archiving [13].

However, existing technologies by which high-resolution spectra are directly measured [14,15,16] often suffer from physical bulkiness, restricted mobility, poor light sensitivity and/or long capturing time. For fast and less costly alternatives, using compressed sensing, the spatial and spectral information is jointly encoded in the captured 2D images and decoded by specialized algorithms [17,18,19,20,21,22,23]. Most of these approaches use learning algorithms to solve for the complex and ill-posed decompression.

As one of the learning approaches, spectral reconstruction (SR) seeks to reconstruct hyperspectral information from spectral images of fewer spectral channels. While many works in the literature propose ways to increase the number of captured spectral channels—including using a multispectral color filter array [24,25,26], a color filter wheel [27], multiple RGB cameras [28], multiple LED light sources [29,30], a stereo camera [31] and faced reflectors [32]—there are many works that focused on recovering hyperspectral information from the RGB images of a single camera [33,34,35,36,37,38,39,40,41,42,43,44,45,46,47,48,49,50,51]. Indeed, research has shown that in a natural scene a significant portion of spectral variation is captured by its color appearance [52], which makes it possible for learning approaches to infer much spectral information from the RGB data. Moreover, spectral recovery might be further improved when colors are part of the spatial context (the patterning of RGB colors), e.g., [47,48].

In this paper, we concern ourselves with the physical plausibility of SR from RGB images. Clearly, spectra and RGBs are physically related: an RGB camera integrates spectra with the spectral sensitivities of three different color sensors, resulting in the 3-value RGB colors, yet this physical fact is generally not employed by the best SR algorithms. Indeed, it is shown in [48] that the top deep learning approaches [50,53] recover spectral estimates that do not physically induce the same RGBs. This color fidelity issue is of practical importance. For instance, in some applications where color accuracy is concerned (e.g., art archiving), we clearly do not wish to use an algorithm that cannot regenerate the original colors.

**Figure 1:**
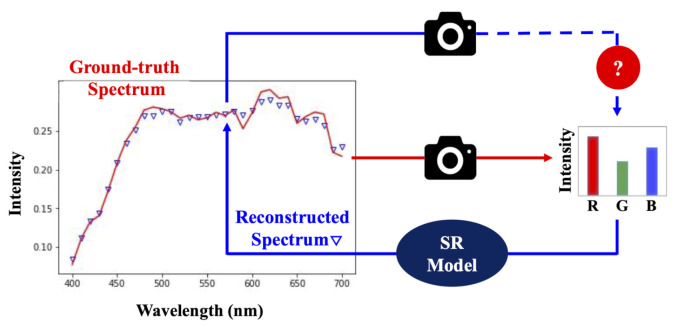
Our physical plausibility (color fidelity) test for SR.

**Figure 2:**
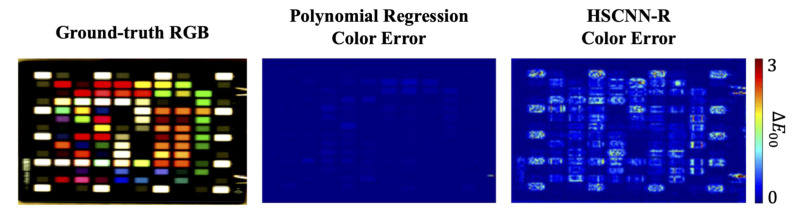
The color errors introduced by polynomial regression SR [34] (**left**) and HSCNN-R [46] (**right**). The color errors are measured in CIE ΔE 2000 ($\Delta E_{00}$) [54].

Figure 1 illustrates our physical plausibility test for SR. While the ground-truth RGBs can be generated from the hyperspectral data (red curve), we test the color fidelity of the reconstructed spectra (blue dotted curve—estimated by each tested SR algorithm) when reintegrated with the same set of spectral sensitivities. In Figure 2, we give an example of the color errors introduced by polynomial regression SR [34] and one of the leading deep-learning models, HSCNN-R [46]. We can clearly see that HSCNN-R—despite claiming the state-of-the-art spectral accuracy [47]—performs much worse in color than the regression-based polynomial regression model. However, the very existence of the non-zero color errors indicates that “both methods are physically implausible”.

**Figure 3:**
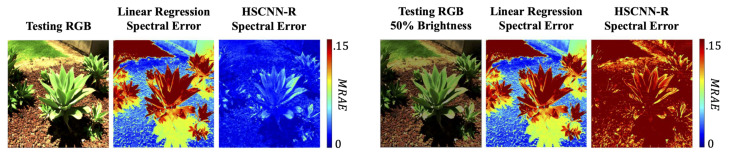
Spectral reconstruction under varying exposure by linear regression [33] and HSCNN-R [46]. The spectral errors are calculated in mean relative absolute error (MRAE) [47,48].

The second problem inherent in the current state of the art is exposure invariance. There are several factors that can result in exposure change, including there being more or less light; the same object being viewed in different parts of the scene (and being recorded as brighter or darker); or the device itself might globally or locally change its exposure setting (e.g., the EV value). In this case, physically, the corresponding spectra are a scalar scaling apart [35]. That is, the magnitude of the physical spectrum changes but its shape remains the same. This said, we might expect that, for the two RGBs from the same but differently exposed physical object, the SR algorithms should recover two spectra with a scaling apart. Unfortunately, this is normally not the case. It is shown in [35] that the leading deep learning methods only work for a fixed exposure; i.e., the shape of the recovered spectra also changes with exposure. Moreover, the change in shape can be surprisingly large. The example in Figure 3 exhibits the extent of deterioration as we seek to reconstruct spectra from a 50% dimmer RGB image using the HSCNN-R model, in comparison to the primitive but exposure-invariant linear regression SR [33].

In this paper, we extend the existing SR algorithms to ensure that they return physically plausible spectra and that they continue to work well when the exposure changes. To solve the physical plausibility issue (to make the recovered spectra reintegrate to the same initial RGBs), our insight is to represent the output spectral space of the SR algorithm by the sum of the unique fundamental metamer of the given RGB and a non-unique metameric black. The fundamental metamer is in the space spanned by the spectral sensitivities of the camera and the metameric black is orthogonal to the spectral sensitivities. We reformulate SR estimation so that the reconstruction adheres to the fundamental metamer plus metameric black decomposition. In effect, we change the estimation problem from one of recovering the “most likely spectrum” to recovering the “most likely metameric black”. Importantly, our method can be directly implemented in all methodologies, including deep learning.

Our solution to stabilizing the models under varying exposure is more pragmatic. We randomly modulate the exposure of the data (i.e., spectra and corresponding RGBs) when training the models. This simple data augmentation approach can make a dramatic difference in the accuracy of recovered spectra when the exposure changes.

We tested our methods on both the regression-based models (which includes the leading sparse coding and a shallow network solution) [33,34,35,36,37] and an exemplar leading deep neural network (DNN) model [46]. Experiments show that we can ensure the physical plausibility of the recovered spectra without negatively affecting recovery performance. Additionally, incorporating exposure variation in training leads to a significant uplift in recovery performance when exposure changes.

**Figure 4:**
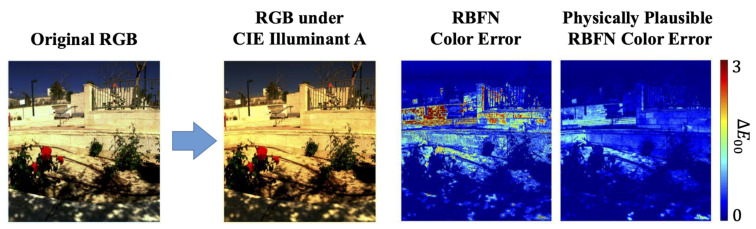
The scene relighting color fidelity of one example hyperspectral image recovered by the RBFN algorithm [36] and by our physically plausible modification of RBFN. The results are shown as the error maps of CIE ΔE 2000 color differences ($\Delta E_{00}$) [54].

Finally, since we are recovering spectra which can be physically projected to the desired RGBs, this means we can change the illumination spectra and/or the camera’s spectral sensitivities and get new RGBs for another viewing condition. We present experiments which demonstrate that a physically plausible spectral recovery results in better cross-viewing-condition color prediction (Figure 4 shows an example of the cross-illumination color fidelity result when using our physically plausible approach).

## 2. Background

Spectral reconstruction (SR) has been intensively studied in both the color science and computer vision communities. Maloney and Wandell [38] represented reflectances using a 3-dimensional linear model. With respect to this model the spectra are related to RGBs by a simple 3×3 matrix transform. Additionally, RGBs for the same surfaces viewed under a pair of different lights must be a 3×3 linear transform apart. However, several subsequent studies showed that to adequately represent spectra, a higher than 3-dimensional linear model is required [55,56,57,58,59]. For higher-dimensional models the spectral reconstruction problem is ill-posed. Indeed, so long as the model has four or more degrees of freedom, we can always find (e.g., using “singlar value decomposition”, referring to pp. 382–391 in [60]), one or more axes in the spectral space that are orthogonal to the spectrum-to-RGB projection. Since the values along these axes do not influence the resulting RGB values (i.e., the same RGB can be derived from different spectra with differences only in these axes), there must be a set of infinite spectra—called the metamers [61]—corresponding to one given RGB. In this paper we say the metamers belong to the plausible set of a given RGB.

Spectral recovery in the ill-posed case seeks to find the most likely spectrum for a given RGB. Recovery methods range from simple statistical approaches, including least-squares regression [33,34,35], Bayesian approaches [41,42] and iterative methods [43,44], to data clustering-based algorithms, such as the radial basis function network [36] and sparse coding [37,45], to the newest deep neural networks (DNN) [46,47,48,49,50,51].

A key seductive argument made about DNN approaches is that—perhaps at an object description level—a pixel is viewed in the context of an image, which helps determine the object and hence the shape of the spectrum. This idea clearly has some merit. After all, almost all cameras now automatically find faces in images, and the reflectance of skin has a characteristic spectral shape [62,63]. However, in experiments—as per [37] and the results presented in this paper—DNNs deliver only a modest performance increment compared to simpler methods.

Providing some motivation for the approach we develop in this paper, there were already studies that used the physics of image formation to improve spectral reconstruction. Agahian et al. [39] proposed to characterize each 3-dimensional reflectance dynamically with emphasis on the reflectance data of close-by colors. Zhao et al. [40] developed a matrix-R approach to colorimetrically post-facto correct the linear regression-based SR. Morovic and Finlayson [42] used metamer sets [61] as the physical constraints of Bayesian inference (and recovered spectra that are physically plausible). However, the performance of that method—developed over 10 years ago—is not competitive with today’s leading methods. Bianco [43] proposed an iterative algorithm which includes color difference in the optimization function. Most recently in the NTIRE 2020 Spectral Recovery Challenge [48], the first-place winner Li et al. [50] included color difference in their learning cost function, and Joslyn Fubara et al. [51] designed an unsupervised learning approach based on the physics prior. However, even these last two methods still recover physically implausible spectra (spectra of wrong colors) [48].

### 2.1. Image Formation

The radiance spectrum is an intensity distribution across wavelengths, denoted as a spectral function r(λ). Correspondingly, the R, G and B sensors are characterized by spectrally-varying sensitivities, denoted as $s_{k}\left(\lambda \right)$ with k=R,G,B. Based on this nomenclature, the RGB image formation is written as [64]:

(1) $$\int_{\Omega }s_{k}\left(\lambda \right)r\left(\lambda \right)d\lambda =\rho_{k}\;,$$

where Ω refers to the visible range, which is set to [400,700] nanometers in this paper, and $\rho_{k}$ is the color value in the *k* channel.

In reality, the ground-truth spectra are measured discretely—at *n* evenly spaced wavelengths—by hyperspectral cameras. Hence, one can vectorize Equation (1):

(2) $$S^{T}\underline{r}=\underline{\rho }\;,$$

where $\underline{r}\in R^{n}$ is the discrete representation of spectra, $S=({\underline{s}}_{R},{\underline{s}}_{G},{\underline{s}}_{B})$ is the n×3 spectral sensitivity matrix and $\underline{\rho }={\left(R,G,B\right)}^{T}$ represents the 3-value RGB color. This $\underline{\rho }$ vector refers to the linear color or raw camera response, which is commonly used as ground-truth RGBs for training the SR algorithms, e.g., in [36,37,45] and the “clean track” in the yearly NTIRE Spectral Recovery Challenge: [47,48]. Essentially, this simple model depicts the physical relationship between the RGBs and spectra.

### 2.2. Spectral Reconstruction

Spectral reconstruction algorithms map RGB colors to the spectral estimates. If we denote an SR algorithm as a mapping function $\Psi :\;R^{3}\mapsto R^{n}$, SR can be simply expressed as:

(3) $$\Psi \left(\underline{\rho }\right)\approx \underline{r}\;.$$

For DNN algorithms, a spectrum is recovered given the image context. Let us denote the set of proximal pixels to $\underline{\rho }$ as $Prox(\underline{\rho })$. A more general form of spectral reconstruction is then written as:

(4) $$\Psi \left(\underline{\rho };Prox\left(\underline{\rho }\right)\right)\approx \underline{r}\;.$$

Equation (4) makes the dependence on context explicit; we will henceforth—to simplify the notation–denote SR algorithms using the notation $\Psi (\underline{\rho })$. In all cases the more general form of SR can be substituted without changing any argument made on our part.

#### 2.2.1. Spectral Reconstruction by Regression

Many algorithms for spectral recovery can be formulated as regressions (linear or non-linear). The standard formulation of the regression-based SR is written as [33]:

(5) $$\Psi \left(\underline{\rho }\right)=M\varphi \left(\underline{\rho }\right)\approx \underline{r}\;,$$

where $\varphi :R^{3}\mapsto R^{p}$ is a bespoke feature mapping for each algorithm, and M is called the regression matrix, which linearly maps the *p*-dimensional features to spectra. If spectra are represented by *n* numbers, then M is an n×p matrix. Recasting Equation (5) as an optimization, the least-squares regression-based SR seeks the M that minimizes:

(6) $$\min_{M}\sum_{i}||M\varphi \left({\underline{\rho }}_{i}\right)-{\underline{r}}_{i}{\left|\right|}^{2}\;,$$

where here—and throughout this paper—$\left|\right|\cdot {\left|\right|}^{2}$ denotes the sum-of-squares or the squared Frobenius norm. Here, *i* indexes over the training set of paired RGBs and corresponding ground-truth spectra.

Let us consider the meaning of $\varphi (\underline{\rho })$. For linear regression [33], $\varphi \left(\underline{\rho }\right)=\underline{\rho }$, i.e., it is the identity transform. For polynomial regression [34] and root-polynomial regression [35], the φ functions are respectively the polynomial and root-polynomial expansions of $\underline{\rho }$ up to a given order. Another non-linear model is the radial basis function network (RBFN) [36], where φ corresponds to the set of outputs from the radial basis functions centered at a given number of representative RGBs. This model is often seen as a shallow neural network solution (consisting only one hidden layer, compared to significantly more for DNNs).

The leading sparse coding algorithm, A+, is also regression based (which is shown to deliver performance close to the DNN solutions) [37]. In sparse coding, we assume that all spectra can be represented as a convex combination of neighboring spectra, and the same combination coefficients will also derive their projected RGBs. In A+, a fixed set of anchor spectra and RGBs are determined by K-SVD clustering [65] (from which the clusters’ centers are selected). Then, around each of the anchor spectra, a given (fixed) number of nearest neighbors are used to solve a linear map (i.e., $\varphi \left(\underline{\rho }\right)=\underline{\rho }$), which is the same as the linear regression model but with only the neighboring data. In reconstruction, the nearest anchor RGB of the input RGB is found, and the trained map of that specific anchor is applied to the input RGB to recover spectra.

All regression algorithms are tuned with regularization [33,66], which is a tool for tackling the overfitting problem [67] (the details of regularization theory fall outside the scope of this paper, but the interested reader is pointed to [33,66]).

#### 2.2.2. An Exemplar DNN Algorithm

**Figure 5:**
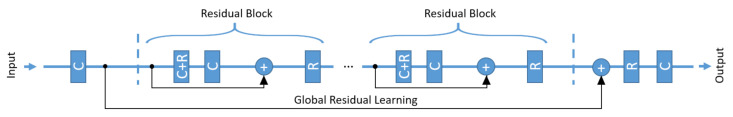
The HSCNN-R architecture [46]. “C” means 3×3 convolution and “R” refers to the ReLU activation.

In Figure 5, we illustrate the HSCNN-R architecture. HSCNN-R [46] was the second-place winner in the 2018 NTIRE Spectral Recovery Challenge [47], and is based on a deep residual learning framework [68]. Each of the residual blocks is constructed with two convolutional layers and one ReLU layer. The model also adopts a global residual learning structure. All convolutional kernels are set to 3×3. In the original setting, the network maps 50×50×3 (height × width × spectral dimension) RGB image patches to the corresponding 50×50×31 hyperspectral image patches (i.e., the ground-truth hyperspectral images used for training have 31 spectral channels). The reader who is interested in how the network is trained is pointed to [46].

## 3. Physically Plausible Spectral Reconstruction

**Figure 6:**
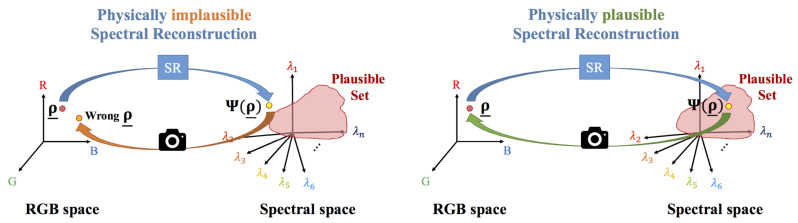
Physically implausible (**left**) and physically plausible spectral reconstruction (**right**).

Figure 6 contrasts physically plausible and implausible spectral recovery. On the left we show implausible spectral reconstruction which represents how many current algorithms work. An image RGB is mapped to a spectrum and this spectrum is almost always outside the plausible set. In this scenario, when the recovered spectrum is integrated with the camera sensors, the resultant RGB is different from the one we started with. On the right of Figure 6, we show physically plausible spectral reconstruction. Here the recovered spectrum is inside the plausible set and so integrates to the same RGB that we started with.

A spectral reconstruction algorithm is said to be physically plausible if and only if for all RGBs (viewed in all contexts), the recovered spectrum integrates to the same RGB:

(7) $$S^{T}\Psi \left(\underline{\rho }\right)=\underline{\rho }\;.$$

Here we adopt the notation introduced in the background section: $\underline{\rho }$, $\Psi (\underline{\rho })$ and S, respectively, denote an RGB, the recovered spectrum (an n×1 vector) and the spectral sensitivities of the camera (an n×3 matrix). We call Equation (7) the color fidelity constraint.

### 3.1. The Plausible Set

Based on the color fidelity constraint, we define the plausible set as all spectra that integrate to the same RGB, which depends on a given RGB and the spectral sensitivities of the camera:

(8) $$P\left(\underline{\rho };S\right)=\left\{\underline{r}\;|\;S^{T}\underline{r}=\underline{\rho }\right\}\;.$$

Let us consider the plausible set in more detail. First we assume that all three sensors—the columns of S—are linearly independent of one another (none can be written as a sum of the other two). In the language of vector spaces, S, is thus a basis defining a 3-dimensional subspace of the *n*-dimensional spectral space. There is a complimentary n×(n−3) basis B whose columns are linearly independent and together span an (n−3)-dimensional subspace of $R^{n}$, and such that $B^{T}S=0$, where 0 is an (n−3)×3 matrix of zeros signifying that B is orthogonal to S. Combined, the n×n matrix [SB] is a basis for the *n*-dimensional space of spectra.

Any given radiance spectrum $\underline{r}$ can be uniquely split into two components: one is the projected component on the basis S, and the other part lies in B:

(9) $$\underline{r}=P^{S}\underline{r}+P^{B}\underline{r}\;,$$

where

(10) $$\left\{\begin{array}{c} P^{S}=S{\left(S^{T}S\right)}^{-1}S^{T}\\ P^{B}=I-P^{S} \end{array}\right.$$

are the n×n projector matrices of S and B, respectively (I is the n×n identity matrix). The significance of “projection” is that $P^{S}\underline{r}$ and $P^{B}\underline{r}$ are respectively, over all other vectors in the span of S and B, closest to the original radiance $\underline{r}$ in a least-squares sense (pp. 219–232; [60]).

Projector matrices have the natural property that their rank is equal to the dimension of the subspace on which they project. Thus, from this projector $P^{B}$ it follows that we can solve for basis B. From elementary linear algebra, we know that $P^{S}$ has rank 3 (since S is 3-dimensional) and $P^{B}$ has the complementary rank n−3 (pp. 135–149; [60]). The basis B is then the n−3 linearly independent columns of $P^{B}$, which can be found using, e.g., the Gram–Schmidt orthogonalization procedure [69].

In Equation (9), the spectral components $P^{S}\underline{r}$ and $P^{B}\underline{r}$ are respectively called the “fundamental metamer” and “metameric black” [70]; henceforth, we denote them as ${\underline{r}}^{f}$ and ${\underline{r}}^{b}$, respectively. Returning to the definition of a plausible set, Equation (8), the color fidelity constraint $S^{T}\underline{r}=\underline{\rho }$ ensures that all spectra $\underline{r}$ in $P(\underline{\rho };S)$ have the same fundamental metamer ${\underline{r}}^{f}$. Indeed, since

(11) $${\underline{r}}^{f}=P^{S}\underline{r}=S{\left(S^{T}S\right)}^{-1}\left(S^{T}\underline{r}\right)\;.$$

It follows:

(12) $${\underline{r}}^{f}=S{\left(S^{T}S\right)}^{-1}\underline{\rho }\;.$$

In other words, ${\underline{r}}^{f}$ can be derived directly from the RGB vector $\underline{\rho }$; therefore, **no** estimation is needed. What is also indicated in Equation (12) is that an RGB has a unique fundamental metamer and vice versa.

Now let us consider the other part of the spectra, the metameric black component ${\underline{r}}^{b}$. ${\underline{r}}^{b}$ lies in the basis B which is orthogonal to S, and when integrated with the spectral sensitivities, induces a zero color response, i.e., $S^{T}{\underline{r}}^{b}=\underline{0}$ (here, $\underline{0}$ is a 3-vector of zeros). Given only the input RGB, it follows that all metameric blacks which lies in B are possible solutions (since it is not constrained by the color fidelity constraint). We can represent the set of all metameric blacks as:

(13) $${\underline{r}}^{b}=B\underline{b}\;,$$

where $\underline{b}$ is an (n−3)×1 coefficient vector.

Based on the derivations above, we write $P(\underline{\rho };S)$ in the form of $\left[{\underline{r}}^{f}+{\underline{r}}^{b}\right]$:

(14) $$P\left(\underline{\rho };S\right)=\left\{{\underline{r}}^{f}+B\underline{b}\;|\;\underline{b}\in R^{n-3}\right\}\;.$$

### 3.2. Estimating Physically Plausible Spectra from RGBs

The aim of spectral reconstruction is to recover a radiance spectrum $\Psi (\underline{\rho })$ from an RGB $\underline{\rho }$ that is as close to the correct answer $\underline{r}$ (the ground-truth) as possible. All algorithms Ψ have tunable parameters that seek to minimize the recovery error: the distance between the recovered spectrum and the ground-truth radiance.

The error between one spectral estimate $\Psi (\underline{\rho })$ and the correct ground-truth $\underline{r}$ is written as:

(15) $$\mathrm{recovery}\;\mathrm{error}=||\;\underline{r}-\Psi \left(\underline{\rho }\right)\;\left|\right|\;.$$

Remember that we are representing a spectrum as a sum of the spectrum’s fundamental metamer and a metameric black: $\underline{r}={\underline{r}}^{f}+{\underline{r}}^{b}$. At the core of our physically plausible SR approach is to derive (instead of estimate) the exact ${\underline{r}}^{f}$ from the RGB i.e., using Equation (12). It follows that the recovery error only depends on how well the ${\underline{r}}^{b}$ part of the spectrum is recovered.

Let us denote an algorithm which recovers the metameric black part of the spectrum as $\Psi^{b}$. Given a set of training spectra and RGBs, ${\underline{r}}_{i}$ and ${\underline{\rho }}_{i}$ (*i* indexes an individual data pair), we seek to minimize:

(16) $$\min_{\Psi^{b}}\;\;\sum_{i}\;||\;{\underline{r}}_{i}^{b}-\Psi^{b}\left({\underline{\rho }}_{i}\right)\;\left|\right|\;,$$

where the ground-truth ${\underline{r}}_{i}^{b}$ can be calculated by the projector matrix ${\underline{r}}_{i}^{b}=P^{B}{\underline{r}}_{i}$. To ensure that $\Psi^{b}$ must recover estimates that lie in basis B, we restrict the estimated metameric black to comply with the linear combination form $\Psi^{b}\left({\underline{\rho }}_{i}\right)=B{\underline{b}}_{i}$ (Equation (13)). Equation (16) can then be, equivalently, written as:

(17) $$\min_{{\underline{b}}_{i}}\;\;\sum_{i}\;||\;{\underline{r}}_{i}^{b}-B{\underline{b}}_{i}\;\left|\right|\;.$$

Counterintuitively, Equations (16) and (17) teach that the physically plausible spectral recovery involves estimating the part of radiance that a camera cannot see.

**Figure 7:**
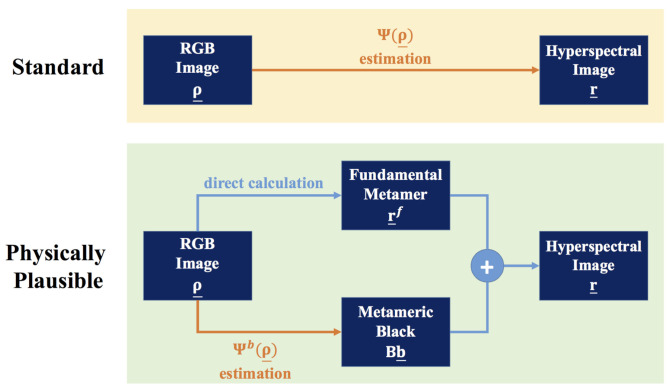
The standard SR scheme (**top**) versus our physically plausible SR scheme (**bottom**).

In Figure 7, we compare our physically plausible method with the conventional approach (which does not recover physically plausible spectra). In the standard approach (top of the figure) the training/estimation scheme directly maps the RGBs to spectra. Here, $\underline{r}$ may not integrate to $\underline{\rho }$ (the RGB from which it was recovered). In the physically plausible approach, the reconstruction is split into two streams. In the first stream the fundamental metamer—which is the only part that contributes to the RGB formation—is calculated directly from the input RGB. Then, the second stream seeks to find the best estimate for the metameric black. By construction the recovered spectrum (the sum of the fundamental metamer and the metameric black) must integrate to the same RGB.

#### 3.2.1. Physically Plausible Regression-Based Models

In the case of regression, we return to the formulation of regression-based SR in Equations (5) and (6). We now in turn solve for the map from the RGB—or more generally from its feature expansion $\varphi (\underline{\rho })$—to the metameric black. With $\Psi^{b}\left(\underline{\rho }\right)=M^{b}\varphi \left(\underline{\rho }\right)$, we minimize:

(18) $$\min_{M^{b}}\;||M^{b}\varphi \left(\underline{\rho }\right)-{\underline{r}}^{b}\left|\right|\;.$$

Further, according to Equation (17) we have to constrain $\Psi^{b}\left(\underline{\rho }\right)$ such that it only recovers metameric black. It follows that we can decompose $M^{b}$ into:

(19) $$M^{b}=BM\;,$$

where M is an (n−3)×p matrix (remember B is the n×(n−3) orthogonal basis spanning the set of metameric blacks). Then, we can rewrite Equation (18) as:

(20) $$\min_{M}\;||BM\varphi \left(\underline{\rho }\right)-{\underline{r}}^{b}\left|\right|\;.$$

Since B is an orthogonal matrix, we know that $B^{T}B=I$ and $\left||A|\right|=\left|\right|B^{T}A\left|\right|$ for any arbitrary matrix A. Hence,

(21) $$||BM\varphi \left(\underline{\rho }\right)-{\underline{r}}^{b}\left|\right|=\left|\right|B^{T}\left(BM\varphi \left(\underline{\rho }\right)-{\underline{r}}^{b}\right)\left|\right|=||M\varphi \left(\underline{\rho }\right)-B^{T}{\underline{r}}^{b}\left|\right|\;.$$

Finally, the physically plausible spectral recovery as a regression problem sets out to find the M that minimizes this norm.

#### 3.2.2. Physically Plausible Deep Neural Networks

Likewise for the DNNs, we can replace the regression mapping $M\varphi (\underline{\rho })$ in the above discussion by a DNN model such that

(22) $$DNN\left(\underline{\rho }\right)\approx B^{T}{\underline{r}}^{b}\;,$$

that is, to modify the original DNN algorithm to estimate $B^{T}{\underline{r}}^{b}$ instead of spectra. Following the same logic in Equations (18) and (19) we have

(23) $$\Psi^{b}\left(\underline{\rho }\right)=BDNN\left(\underline{\rho }\right)\approx {\underline{r}}^{b}\;,$$

which recovers the metameric black.

However, for many DNN models (including the one considered in this paper), the output layer is constricted to return positive values (since its original usage is to recover all positive spectra), and yet $B^{T}{\underline{r}}^{b}$**must** have some values that are negative. For this reason we investigated the range of $B^{T}{\underline{r}}^{b}$ in our testing dataset. Assume that the maximum value in the original hyperspectral images is $v_{\max}$ (e.g., in our case the images are 12-bit, so $v_{\max}=2^{12}-1=4095$), empirically, we found that $B^{T}{\underline{r}}^{b}$ are bounded by $\left[-v_{\max},v_{\max}\right]$. Without changing the original model, we set the DNN algorithm to recover instead the offset values $\frac{1}{2v_{\max}}\left(B^{T}{\underline{r}}^{b}+v_{\max}\right)$, which is then corrected back to $B^{T}{\underline{r}}^{b}$ after reconstruction.

### 3.3. Intensity-Scaling Data Augmentation

The same object viewed in different parts of the same scene or viewed under different intensities of light and/or different camera exposure settings can appear brighter or darker. The brightness change due to there being more or less light is called a change of exposure. Let us model exposure change by a scaling factor *k* multiplying the radiance spectrum: $\underline{r}\to k\underline{r}$. Clearly, according to Equation (2) the corresponding RGB is scaled by the same factor:

(24) $$S^{T}\left(k\underline{r}\right)=k\left(S^{T}\underline{r}\right)=k\underline{\rho }\;.$$

Unfortunately, as shown in [35,48], in most existing algorithms—and all of the leading DNN-based spectral reconstruction approaches,

(25) $$\Psi \left(k\underline{\rho }\right)\neq k\Psi \left(\underline{\rho }\right)\;.$$

That is, the shape of the recovered spectrum changes as the exposure changes (not just its magnitude as prescribed by the physics).

Our solution to this problem is pragmatic. Given a pair of RGB and spectrum for training, $\left({\underline{\rho }}_{i},{\underline{r}}_{i}\right)$, we multiply them with a random scaling factor *k*, such that $\left(k{\underline{\rho }}_{i},k{\underline{r}}_{i}\right)$ is used as a replacement of the original pair in training. We, of course, must use many different scaling factors (for different training pairs). We argue that the random distribution of *k* should follow a uniform distribution on a log scale:

(26) $$\log_{\beta }k∼Uniform\left(-1,1\right)\;,$$

where β controls the range of the distribution, e.g., for β=10, the distribution is bounded by $\left[\frac{1}{10},10\right]$.

**Figure 8:**
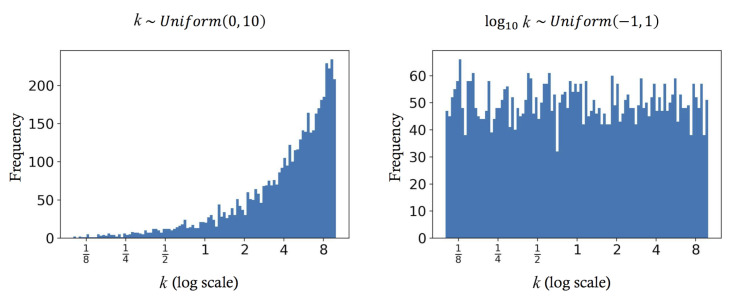
The comparison between drawing the scaling factor *k* from the straightforward uniform distribution (**left**) and from our proposed distribution (**right**).

The justification of using this random distribution is demonstrated in Figure 8. Let us compare the proposed distribution (β=10; right panel) with the straightforward uniform distribution between [0,10] (left panel). From both distributions we drew 5000 random numbers and show the histogram with 100 bins on the log scale (linear to the “geometric progression” of the exposure modes of a usual imaging device). Evidently, the straightforward uniform distribution generates exponentially more bright scaling factors than the dim ones, while our proposed distribution provide equal chances for bright and dim factors to be chosen.

For the regression-based models, we simply apply the random scaling factors to all individual pairs of spectrum and RGB prior to the training. Now for the DNN model we implement data augmentation slightly differently. By virtue of the iterative training process of DNN, instead of generating all augmented data before training, we apply the random scaling factors in real-time—different image patches and the same patches in different training epochs are applied with different scaling factors. This setting in effect provides far more chances for the model to see the introduced exposure variation.

Another implementation detail is that, once we allow different exposure scaling factors, we essentially stretch the range of the output space of the physically plausible DNN to $\left[-\beta v_{\max},\beta v_{\max}\right]$ (which was originally $\left[-v_{\max},v_{\max}\right]$; see the discussion in Section 3.2.2). Hence, in our case that the considered DNN model only allow positive output values, we need to apply an offset following: $\frac{1}{2\beta v_{\max}}\left(B^{T}{\underline{r}}^{b}+\beta v_{\max}\right)$.

## 4. Experiments

In Table 1, we list six exemplar algorithms we tested (see table for algorithm names and abbreviations), which comprise five regression-based algorithms and one exemplar DNN approach (these algorithms are reviewed in Section 2). According to [35], LR, RPR and A+ are exposure-invariant, which means they perform equally well for a varying exposure as they do for fixed exposure conditions. This means we did not need an additional data augmentation process (detailed in Section 3.3) to ensure their generalizability to different exposure conditions.

We will compare spectral recovery for all considered algorithms where a standard training methodology is used (color fidelity is not enforced) and with our new physically plausible SR formulation (that guarantees color fidelity). All implemented codes are provided as the Supplementary Materials.

### 4.1. Image Dataset

**Figure 9:**
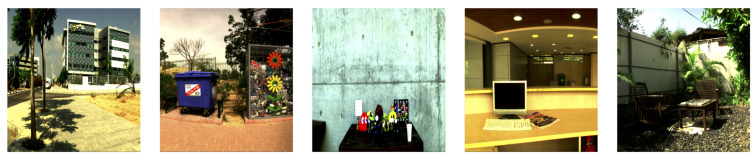
Example scenes from the ICVL hyperspectral image database [45].

In this paper, we used the ICVL database [45], which consists of 201 hyperspectral images of both indoor and outdoor scenes. The spatial dimensions of the scenes are 1300×1392, and the spectra were measured from 400 to 700 nanometers (nm) with 10-nm intervals, resulting in 31 spectral channels. All values of the images are encoded in 12 bits. Some example scenes from the database are shown in Figure 9.

Then, we simulated the ground-truth RGB images following the linear RGB image formation (Equation (2)), with the CIE 1964 color matching functions (CMF) [71] as the camera’s spectral sensitivities. This choice of using CMF is so we follow the standard methodology of the yearly NTIRE competition on spectral recovery [47,48]. We also remark that the CIE 1964 CMF is a revised version of the CIE 1931 CMF [72], which addressed the influence within the 10${}^{\circ }$ viewing angle of the standard observer, as opposed to the 2${}^{\circ }$ viewing angle considered in CIE 1931 CMF.

### 4.2. Cross Validation

In this paper we use a 4-trial cross validation setting. We randomly allocate all images into four groups—conceptually, group *A*, *B*, *C* and *D*. We designed a compact 4-trial setting:

- Trial 1—Train set: A+B, Validation set: *C*, Test set: *D*,
- Trial 2—Train set: A+B, Validation set: *D*, Test set: *C*,
- Trial 3—Train set: C+D, Validation set: *A*, Test set: *B*,
- Trial 4—Train set: C+D, Validation set: *B*, Test set: *A*.

In each trial, two groups of images were used for training, one group for validation and one group for testing. Note that for regression-based methods the model validation refers to selecting proper regularization parameters to fit the validation set images (we point the interested readers to [34,35,37] for the implementation details), whereas for the deep learning model we used the validation set data to determine the terminating epoch in the iterative training process. The cross-validated error statistics are then the averaged testing performance over the four trials.

### 4.3. Evaluation Metrics

#### 4.3.1. Spectral Difference

In this paper, we use the following metrics to measure the spectral error. Given a pair of ground-truth spectrum $\underline{r}$ and reconstructed spectrum $\hat{\underline{r}}$:

- Mean relative absolute error:
  (27) $$\mathrm{MRAE}\;\left(\%\right)=100\times \frac{1}{n}||\frac{\underline{r}-\hat{\underline{r}}}{\underline{r}}|{|}_{1}\;,$$
  where *n* is the number of spectral channels (in our case n=31), the division is element-wise and the L1 norm is calculated. Essentially, this MRAE metric measures the averaged percentage absolute deviation over all spectral channels. This metric is regarded as the standard metric to rank and evaluate SR algorithms in the recent benchmark [47,48].
- Goodness of fit coefficient:
  (28) $$\mathrm{GFC}=\frac{\underline{r}}{\left|\right|\underline{r}\left|\right|}\cdot \frac{\hat{\underline{r}}}{\left|\right|\hat{\underline{r}}\left|\right|}\;,$$
  where the inner product of the normalized spectra is calculated. According to [56], acceptable reconstruction performance refers to GFC ≥0.99, GFC ≥0.999 is regarded as very good performance and GFC ≥0.9999 means nearly exact reconstruction.
- Root mean square error:
  (29) $$\mathrm{RMSE}=\sqrt{\frac{1}{n}\left|\right|\underline{r}-\hat{\underline{r}}{\left|\right|}_{2}^{2}}\;,$$
  where *n* is the number of spectral channels. Note that RMSE is scale dependent, that is, the overall brightness level in which the compared spectra reside will reflect on the scale of RMSE. Thus, bear in mind that the images in the ICVL database [45] use 12-bit encoding (i.e., all values are bounded by [0, 4095]) when interpreting the presented results.
- Peak signal-to-noise ratio:
  (30) $$\mathrm{PSNR}=20\times \log_{10}\left(\frac{v_{\max}}{\mathrm{RMSE}}\right)\;,$$
  where $v_{\max}=2^{12}-1=4095$ is the maximum possible value for 12-bit images. Similarly to RMSE, PSNR is scale dependent.

#### 4.3.2. Color Difference

In addition to the spectral error measures, we pay special attention to the models’ colorimetric performances. We used the CIE ΔE 2000 color difference formula ($\Delta E_{00}$) [54] to measure the difference between the ground-truth and reconstructed colors. The implementation of $\Delta E_{00}$ is rather complex: we refer the readers to [54] for details. Practically, a $\Delta E_{00}$ equaling 1 between two color stimuli correlates with a color difference that is just noticeable to a human observer.

Note that the $\Delta E_{00}$ is defined upon the CIELAB [73] color coordinates—one of the standard (device independent) color spaces [74]. From our ground-truth color space, CIEXYZ, there exists direct transformation to CIELAB given a ground-truth white point color (i.e., the illumination color) [74]. In our experiments, we obtained this white point information by hand-crafting the “brightest near-achromatic spectrum” from each ground-truth hyperspectral image and then integrating this white-surface radiance spectrum with the CIE 1964 XYZ color matching functions.

## 5. Results

### 5.1. Effectiveness of Data Augmentation

We can only create the augmented data within a given “range” of exposure variation (it is not feasible to include all possible exposure changes, since the physical brightness level is unbounded). Returning to the random distribution used in our data augmentation approach (Section 3.3; Equation (26)), the range of the random scaling is bounded by $\left[\frac{1}{\beta },\beta \right]$. Clearly, if we choose a larger β, the trained models will have a wider range of generalizability in terms of exposure change. Note that we simulated the scaled images in floating point numbers (no darkened pixels were digitized to 0), and we allowed values exceeding the camera’s original dynamic range (brightened pixels were not clipped at $v_{\max}$). Under this setting, assuming that there is no under- or over-exposed image in the database, there will also not be any of such images among our brightened/darkened images.

**Figure 10:**
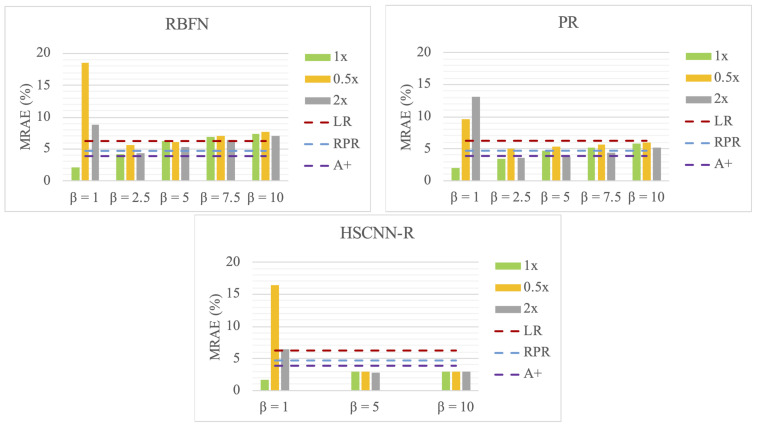
Visualizing the performance and generalizability (in mean MRAE) with respect to different β factors chosen.

In Table 2 and Figure 10, we show how the value of β influences the models’ performance and generalizability. We trained the models with β=1 (i.e., the original training regime), 2.5,5,7.5 and 10 (for the deep learning based HSCNN-R we only trained for β=1,5 and 10). Under this training arrangement, we tested the models with all testing images scaled by factors of 1 (the original images), 0.5 (half exposure) and 2 (double exposure), denoted as “**1x**”, “**0.5x**” and “**2x**”, respectively. The performances of the exposure-invariant models are also given on top of each result table as baselines for comparison, and plotted as dotted lines in the figures.

Notice that here we only present the results in MRAE, GFC and $\Delta E_{00}$. In [37,45,47], it is argued that RMSE tends to penalize bright pixels more than the dim pixels. We remark that this originates from that RMSE, as we mentioned in Section 4.3.1, is scale dependent. Indeed, if we scale both $\underline{r}$ and $\hat{\underline{r}}$ by 2, the RMSE will also be doubled. It is therefore not suitable to use RMSE for comparing the reconstruction results in different exposure scales. The same argument also applies to PSNR.

First, we see that RBFN, PR and HSCNN-R trained under the original training regime (β=1) deliver superior performance in spectral accuracy compared to those exposure-invariant baseline models when tested with the original testing images (**1x**), but deteriorate under other exposure conditions (much worse than the simplest LR model). This result implies that the images (used for training and testing) in the ICVL database [45] were captured under very similar exposure conditions. Granted, when capturing images we often adjust the exposure settings of the device to fit the dynamic range of the scene, so as to avoid over- and under-exposed images, but in doing so we are in effect training the models only to work on those “nicely captured” scenes—say, if a sudden strong light occurs in the scene (e.g., the cars’ headlights) and the rest of the scene darkens for fitting the new dynamic range, the models may not work even for the parts of the image that are not over-exposed.

Through our data augmentation, β of higher values stabilizes the models’ performances in both spectral and color accuracy—though at the cost of worse overall spectral accuracy. Indeed, the performances of the data-augmented RBFN and PR became worse than the baseline models in some cases, while the data-augmented HSCNN-R still held some advantage over the baseline models.

For HSCNN-R, the selection of β does not have much influence on the models’ performance. In contrast, for both RBFN and PR, large β values lead to performance degradation, to the point that the performance can be much worse than the baseline models. As a result, in the forgoing discussion, we select β=2.5 for RBFN and PR, and β=10 for HSCNN-R.

Notice that the HSCNN-R with data augmentation clearly delivers good generalizability for the three testing exposures (i.e., small differences between the **1x**, **0.5x** and **2x** results). On the other hand, despite improvement, both RBFN and PR only exhibit limited generalizability. Indeed, for both models the performance for the **0.5x** condition is generally worse than that for the **1x** and **2x** exposure. We note that the powerful HSCNN-R has many more parameters than the polynomial or RBF regressions (so it is not entirely surprising that the DNN model improves more significantly given the augmented training data).

Another interesting phenomenon can be viewed in the results is that: the spectral accuracy does not imply color accuracy. We see from the mean $\Delta E_{00}$ results, the most primitive LR—albeit much less accurate in spectra—is much accurate in colors than other more complicated models, including RBFN and HSCNN-R (with or without data augmentation). As commented in Figure 2, all presented models are nonetheless physically implausible due to the non-zero color errors.

### 5.2. Effectiveness of Physically Plausible Spectral Reconstruction

#### 5.2.1. Color Fidelity and Spectral Accuracy

In Table 3, we present the effectiveness of physically plausible SR in color and spectral accuracy. Under the “Original” headings, we show the results of the original models (those found in the original citations), and under the “Physically Plausible” headings we present the results of the physically plausible version of the models. For all presented metrics, we calculated the mean and worst-case (99.9 percentile error) of each test image, and then averaged them over all testing images. In continuation of the analysis of the exposure invariance presented in the previous section, we also present the performance of physically plausible SR under varying exposure conditions (i.e., **1x**, **0.5x** and **2x**) in Table 4.

First, let us consider color accuracy. Looking at the error statistics of $\Delta E_{00}$ in Table 3, it is clear that our physically plausible approach forces all models to recover spectra of the exact same colors as the ground-truth—thus, the 0 color error under all circumstances. Then, the spectral accuracy results in all four spectral metrics illustrate that there is no penalty to physically plausible SR (note that for $\Delta E_{00}$, MRAE and RMSE, the lower the numbers are the better, while for GFC and PSNR, the higher the better). Indeed, on average (despite few cases of disagreements among different metrics) enforcing physical plausibility results in a small increase in mean performance. These results indicate that we can, in effect, recover spectra of perfect color fidelity without deteriorating the spectral accuracy. For visualized results, see Figure 13.

Finally, let us look at Table 4. The implementation of physically plausible SR does not influence very much how the models react to exposure change. Indeed, LR, RPR and A+ are still exposure invariant, while RBFN${}^{\left(\beta \;=\;1\right)}$, PR${}^{\left(\beta \;=\;1\right)}$ and HSCNN-R${}^{\left(\beta \;=\;1\right)}$ are not. Additionally, the effectiveness of data augmentation, i.e., RBFN${}^{\left(\beta \;=\;2.5\right)}$, PR${}^{\left(\beta \;=\;2.5\right)}$ and HSCNN-R${}^{\left(\beta \;=\;10\right)}$, still holds for physically plausible SR. Notice that the $\Delta E_{00}$ color error remains zero for all physically plausible models even in the situation that some models’ spectral accuracies deteriorate in varying exposure conditions.

Jointly considering the effectiveness of our proposals—intensity-scaling data augmentation and physically plausible SR—we have achieved SR with no color error and stabilized performance under changing exposure.

#### 5.2.2. Color Fidelity under Different Viewing Conditions

**Figure 11:**
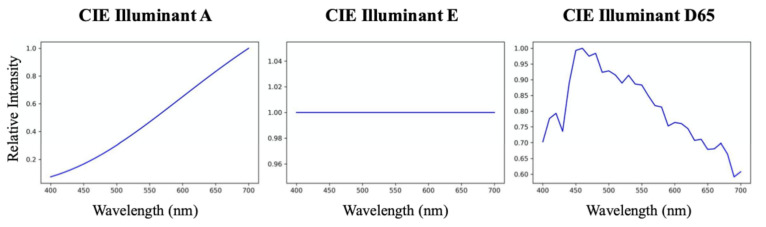
Target illuminants for scene relighting: CIE Illuminants A (**left**), E (**middle**) and D65 (**right**).

**Figure 12:**
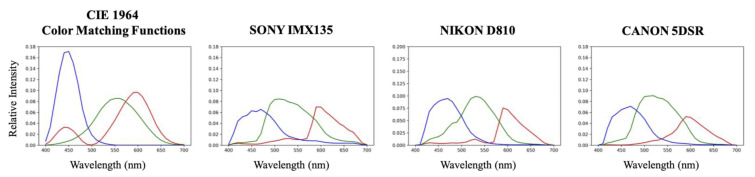
The spectral sensitivities of the ground-truth RGBs used for training (CIE 1964 color matching functions) and for testing (SONY IMX135, NIKON D810 and CANON 5DSR).

Here, we investigate using the hyperspectral recoveries (delivered by the various algorithms) to predict the colors of the same scene under either a different illumination or a different camera. To change the illumination of the scene, first we divide (component-wise) the whole hyperspectral image by the original white spectra and then multiply the image by a target illuminant’s spectrum. Then, this newly derived hyperspectral image can be used to generate the relighted RGB scene using the color formation formula in Equation (2). As for simulating the color responses of a different camera (different from the one that generates the RGBs used to train SR), we need simply to incorporate a different set of spectral sensitivities in Equation (2). The new illumination spectra and camera sensitivities used in the experiments are shown in Figure 11 and Figure 12, respectively.

Note that by relighting the scenes to CIE Illuminant E—which replaces the original illumination spectrum in each image with a “flat” spectral power distribution—effectively, we obtain the new spectra that are (individually) a scaling factor apart from the “reflectance spectra”, which are pure object surfaces’ properties without the influence of the illumination’s spectral property.

**Figure 13:**
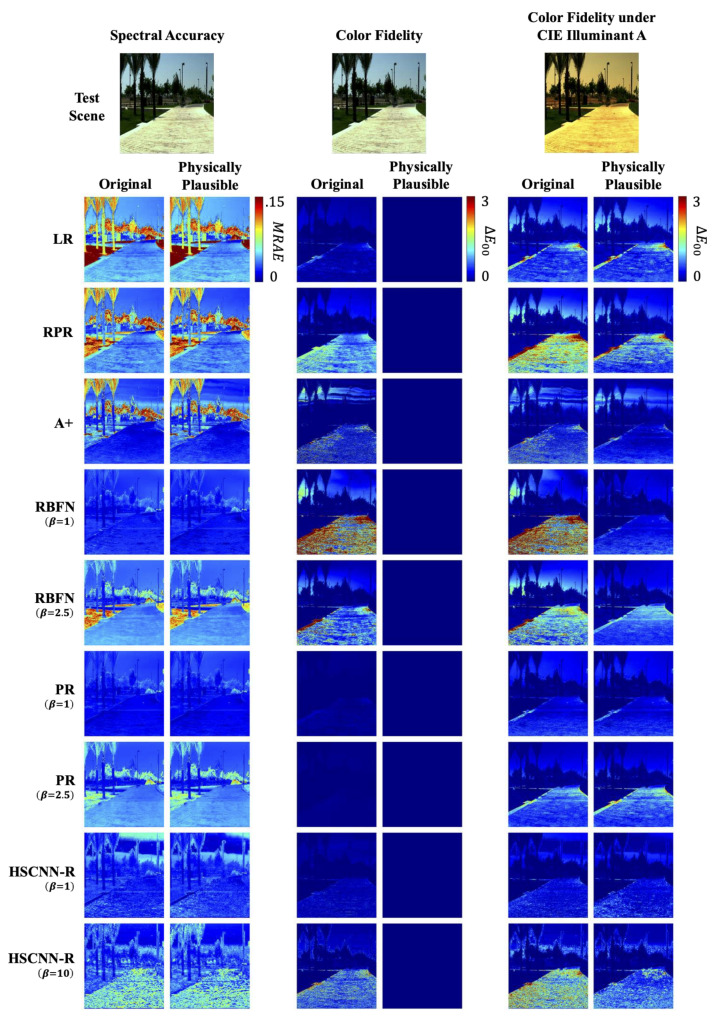
The reconstruction error maps of an example scene in terms of spectral accuracy (**left**; in MRAE), color fidelity (**middle**; in $\Delta E_{00}$) and color fidelity under CIE Illuminant A (**right**; in $\Delta E_{00}$).

We present the color fidelity results of changing the illumination or camera in Table 5. In this experiment we only tested the models under the **1x** (original) exposure condition; i.e., we did not test for exposure variation. Visualized results for CIE Illuminat A relighting can be found in the rightmost column of Figure 13.

First, we see that for all models our physically plausible approach in general improves the cross-illumination and cross-camera color reproduction. If we look at the performances of the original models (without the physically plausible implementation and data augmentation), the PR${}^{\left(\beta \;=\;1\right)}$ model performs the best in predicting the actual cross-illuminant colors, and is up to 30% better than the DNN-based HSCNN-R${}^{\left(\beta \;=\;1\right)}$ model. A similar performance increment is also shown when the camera changes. Ironically, compared to the spectral accuracy results (Table 3), we see PR${}^{\left(\beta \;=\;1\right)}$ recovers spectra that are 13% less accurate in mean MRAE than HSCNN-R${}^{\left(\beta \;=\;1\right)}$. This result tells that while most SR models primarily aim to minimize spectral errors, that does not ensure better performance in the general context of color fidelity, either under the original or changing viewing conditions. Additionally, we showed that as we implement the “physically plausible” HSCNN-R${}^{\left(\beta \;=\;1\right)}$—which actually contributes to improving color fidelity—it is then when HSCNN-R${}^{\left(\beta \;=\;1\right)}$ performs better than PR${}^{\left(\beta \;=\;1\right)}$.

Next, the physically plausible RBFN${}^{\left(\beta \;=\;1\right)}$ exhibited the most improvement from the original model compared to others. Indeed, on average a 60% improvement in cross-viewing-condition color fidelity was delivered by making RBFN${}^{\left(\beta \;=\;1\right)}$ physically plausible. This performance increment also makes it one of the best performing models, on par with PR${}^{\left(\beta \;=\;1\right)}$ and HSCNN-R${}^{\left(\beta \;=\;1\right)}$.

Further, if we consider the effect of data augmentation, we see that—similarly to the spectral accuracy results—RBFN${}^{\left(\beta \;=\;2.5\right)}$, PR${}^{\left(\beta \;=\;2.5\right)}$ and HSCNN-R${}^{\left(\beta \;=\;10\right)}$ in general worsen the performance from that of their original counterparts (β=1). In various circumstances, those data-augmented models deliver much worse mean and worst-case performances compared to the exposure-invariant LR, RPR and A+. Especially, the physically plausible A+ method performs better “in all conditions” than the data augmented physically plausible HSCNN-R${}^{\left(\beta \;=\;10\right)}$. We remind the readers that the exposure-invariant models have the benefit of being able to generalize the exact same performance on the whole scale of physical brightness [35] (e.g., A+), as opposed to the finite range of (often suboptimal) generalizability induced purely by data augmentation (e.g., HSCNN-R).

Given all these experimental results the obvious question to ask is “which algorithm should I choose?” Well, consistent with the trend of adopting DNNs, the HSCNN-R solution—where physical plausibility is enforced and data augmentation is implemented to the training regime—is a good choice overall. However, considering its overhead of long training time and required computing resources, in terms of the various aspects we present in this paper, the exemplar DNN model does not appear to be much superior than the rest of the regression-based methods.

## 6. Conclusions

Spectral reconstruction algorithms seek to map RGB images to hyperspectral images. Most models are designed to minimize the spectral error of the reconstruction, but the underlying physical relationship between spectra and colors is not preserved. This physically non-plausible mapping causes the issues of poor color fidelity and inconsistent performance for the same object viewed at different exposures.

In this paper we provide solutions for both issues. First, we show that all plausible spectra can be represented by a fixed fundamental metamer defined by a linear combination of camera spectral sensitivities, and a metameric black which does not contribute to the color formation. Relative to this insight, the spectral recovery sets out to reconstruct only the metameric black’s coefficients from the RGBs, while the fundamental metamer is derived directly. This ensures that the predicted spectra are always of the exact same RGBs found in the original images. Secondly, we show that better robustness against exposure change can be achieved by augmenting the training data with randomly-generated intensity scaling factors.

Another contribution of this paper is that we performed extensive studies on the models’ colorimetric performances apart from the usual spectral accuracy measure. Our evaluations here included scene relighting and color predictions for different cameras. Our results show that the best performing models—from a color fidelity point of view—do not necessarily correspond to the most spectrally accurate recovery models.

## Figures and Tables

**Table 1:** Exemplar spectral recovery algorithms.

Exposure-Invariant Models	Non-Exposure-Invariant Models
Linear Regression (LR) [33]	Radial Basis Function Network (RBFN) [36]
Root-Polynomial Regression (RPR) [35]	Polynomial Regression (PR) [34]
A+ Sparse Coding (A+) [37]	HSCNN-R Deep Neural Network (HSCNN-R) [46]

**Table 2:** The dependency of spectral and color accuracy on the β factor used for data augmentation. All models were tested under original (**1x**), half (**0.5x**) and double exposure settings (**2x**). The MRAE, GFC and $\Delta E_{00}$ errors are calculated per pixel, and the mean results (over all pixels and images) are shown.

	**Mean MRAE (%) (Spectral Error)**														
	**Baseline Performance: LR = 6.24, RPR = 4.69, A+ = 3.87**														
	β=1			β=2.5			β=5			β=7.5			β=10		
	**1x**	**0.5x**	**2x**	**1x**	**0.5x**	**2x**	**1x**	**0.5x**	**2x**	**1x**	**0.5x**	**2x**	**1x**	**0.5x**	**2x**
RBFN	2.06	18.58	8.74	4.20	5.67	4.33	6.19	6.02	5.30	6.82	7.05	6.40	7.37	7.75	6.98
PR	1.95	9.60	13.04	3.50	5.01	3.57	4.72	5.40	3.80	5.25	5.72	4.45	5.74	6.03	5.13
HSCNN-R	1.73	16.41	6.39	-	-	-	2.91	2.92	2.81	-	-	-	2.96	2.96	2.95
	**Mean GFC (Spectral Error)**														
	**Baseline Performance: LR = 0.9966, RPR = 0.9979, A+ = 0.9983**														
	β=1			β=2.5			β=5			β=7.5			β=10		
	**1x**	**0.5x**	**2x**	**1x**	**0.5x**	**2x**	**1x**	**0.5x**	**2x**	**1x**	**0.5x**	**2x**	**1x**	**0.5x**	**2x**
RBFN	0.9994	0.9802	0.9959	0.9986	0.9981	0.9983	0.9977	0.9979	0.9981	0.9974	0.9971	0.9977	0.9973	0.9968	0.9976
PR	0.9994	0.9949	0.9900	0.9989	0.9984	0.9986	0.9984	0.9981	0.9987	0.9981	0.9979	0.9985	0.9979	0.9977	0.9982
HSCNN-R	0.9995	0.9889	0.9972	-	-	-	0.9992	0.9991	0.9992	-	-	-	0.9991	0.9991	0.9991
	**Mean** $\Delta E_{00}$ **(Color Error)**														
	**Baseline Performance: LR = 0.05, RPR = 0.14, A+ = 0.06**														
	β=1			β=2.5			β=5			β=7.5			β=10		
	**1x**	**0.5x**	**2x**	**1x**	**0.5x**	**2x**	**1x**	**0.5x**	**2x**	**1x**	**0.5x**	**2x**	**1x**	**0.5x**	**2x**
RBFN	0.32	0.68	1.97	0.15	0.17	0.37	0.51	0.61	0.76	0.81	1.03	1.00	0.95	1.24	1.20
PR	0.01	0.02	0.15	0.01	0.03	0.01	0.05	0.06	0.04	0.06	0.09	0.04	0.09	0.11	0.05
HSCNN-R	0.10	0.36	0.16	-	-	-	0.17	0.18	0.18	-	-	-	0.15	0.15	0.15

**Table 3:** The colorand spectral accuracy results as the averaged per-image mean and 99.9th percentile (pt). The results are shown respectively in $\Delta E_{00}$, MRAE, GFC, RMSE and PSNR.

	$\Delta E_{00}$ **(Color Error)**				**MRAE (%) (Spectral Error)**				**GFC (Spectral Error)**			
	**Original**		**Physically Plausible**		**Original**		**Physically Plausible**		**Original**		**Physically Plausible**	
	**Mean**	**99.9 pt**	**Mean**	**99.9 pt**	**Mean**	**99.9 pt**	**Mean**	**99.9 pt**	**Mean**	**99.9 pt**	**Mean**	**99.9 pt**
LR	0.05	0.79	0.00	0.00	6.24	22.45	6.23	22.53	0.9966	0.9770	0.9966	0.9767
RPR	0.14	1.48	0.00	0.00	4.69	24.06	4.60	24.86	0.9979	0.9712	0.9979	0.9640
A+	0.06	2.47	0.00	0.00	3.87	21.06	3.83	20.65	0.9983	0.9770	0.9983	0.9770
RBFN${}^{\left(\beta \;=\;1\right)}$	0.32	9.24	0.00	0.00	2.06	14.44	1.96	13.09	0.9994	0.9852	0.9994	0.9854
RBFN${}^{\left(\beta \;=\;2.5\right)}$	0.15	3.36	0.00	0.00	4.20	17.25	4.15	17.00	0.9986	0.9832	0.9986	0.9834
PR${}^{\left(\beta \;=\;1\right)}$	0.01	0.18	0.00	0.00	1.95	12.84	1.94	12.81	0.9994	0.9841	0.9994	0.9843
PR${}^{\left(\beta \;=\;2.5\right)}$	0.01	0.07	0.00	0.00	3.50	17.95	3.46	18.38	0.9989	0.9814	0.9989	0.9802
HSCNN-R${}^{\left(\beta \;=\;1\right)}$	0.10	2.06	0.00	0.00	1.73	12.10	1.76	12.68	0.9995	0.9864	0.9995	0.9842
HSCNN-R${}^{\left(\beta \;=\;10\right)}$	0.15	2.46	0.00	0.00	2.96	16.14	2.93	21.09	0.9991	0.9841	0.9991	0.9686
	**RMSE (Spectral Error)**				**PSNR (dB) (Spectral Error)**							
	**Original**		**Physically Plausible**		**Original**		**Physically Plausible**					
	**Mean**	**99.9 pt**	**Mean**	**99.9 pt**	**Mean**	**99.9 pt**	**Mean**	**99.9 pt**				
LR	33.26	153.49	33.23	153.35	43.34	30.24	43.36	30.33				
RPR	27.80	167.17	27.49	172.33	45.49	29.93	45.71	29.84				
A+	23.97	161.69	24.36	165.61	48.23	29.79	48.21	29.65				
RBFN${}^{\left(\beta \;=\;1\right)}$	18.30	152.57	17.50	138.23	50.63	31.04	50.98	31.62				
RBFN${}^{\left(\beta \;=\;2.5\right)}$	27.70	142.46	27.24	139.51	45.54	30.84	45.67	31.06				
PR${}^{\left(\beta \;=\;1\right)}$	17.05	142.31	17.06	142.55	50.86	31.72	50.86	31.71				
PR${}^{\left(\beta \;=\;2.5\right)}$	23.88	143.93	23.75	146.78	47.03	31.07	47.10	30.96				
HSCNN-R${}^{\left(\beta \;=\;1\right)}$	16.33	139.58	16.34	137.24	52.34	31.58	52.08	31.70				
HSCNN-R${}^{\left(\beta \;=\;10\right)}$	23.56	167.82	22.67	165.65	49.07	29.47	49.38	29.55				

**Table 4:** The spectral and color accuracy of the “physically plausible” SR under original (**1x**), half (**0.5x**) and double exposure settings (**2x**). The results are shown in mean MRAE, mean GFC and mean $\Delta E_{00}$.

	Mean MRAE (%)			Mean GFC			Mean $\Delta E_{00}$		
	(Spectral Error)			(Spectral Error)			(Color Error)		
	Physically Plausible			Physically Plausible			Physically Plausible		
	1x	0.5x	2x	1x	0.5x	2x	1x	0.5x	2x
LR	6.23	6.23	6.23	0.9966	0.9966	0.9966	0.00	0.00	0.00
RPR	4.60	4.60	4.60	0.9979	0.9979	0.9979	0.00	0.00	0.00
A+	3.83	3.83	3.83	0.9983	0.9983	0.9983	0.00	0.00	0.00
RBFN${}^{\left(\beta \;=\;1\right)}$	1.96	17.6	7.63	0.9994	0.9773	0.9958	0.00	0.00	0.00
RBFN${}^{\left(\beta \;=\;2.5\right)}$	4.15	5.47	4.19	0.9986	0.9982	0.9983	0.00	0.00	0.00
PR${}^{\left(\beta \;=\;1\right)}$	1.94	9.72	13.07	0.9994	0.9948	0.9899	0.00	0.00	0.00
PR${}^{\left(\beta \;=\;2.5\right)}$	3.46	4.93	3.55	0.9989	0.9984	0.9986	0.00	0.00	0.00
HSCNN-R${}^{\left(\beta \;=\;1\right)}$	1.76	15.33	6.39	0.9995	0.9844	0.9972	0.00	0.00	0.00
HSCNN-R${}^{\left(\beta \;=\;10\right)}$	2.93	3.00	2.88	0.9991	0.9991	0.9991	0.00	0.00	0.00

**Table 5:** The color accuracy when changing the illumination (**top**) or camera (**bottom**). The results are shown in the averaged per-image mean and 99.9th percentile (pt) $\Delta E_{00}$.

	$\Delta E_{00}$ (Color Error)											
	CIE Illuminant A				CIE Illuminant E				CIE Illuminant D65			
	Original		Physically Plausible		Original		Physically Plausible		Original		Physically Plausible	
	Mean	99.9 pt	Mean	99.9 pt	Mean	99.9 pt	Mean	99.9 pt	Mean	99.9 pt	Mean	99.9 pt
LR	0.38	3.89	0.38	4.00	0.57	6.58	0.56	6.33	0.49	6.05	0.47	5.67
RPR	0.32	4.89	0.29	4.36	0.51	6.49	0.46	6.26	0.44	5.83	0.39	5.66
A+	0.27	4.90	0.24	4.53	0.40	6.72	0.38	6.02	0.34	6.33	0.31	5.51
RBFN${}^{\left(\beta \;=\;1\right)}$	0.37	10.22	0.16	3.66	0.39	10.67	0.14	3.24	0.38	10.74	0.13	3.18
RBFN${}^{\left(\beta \;=\;2.5\right)}$	0.41	5.80	0.35	3.97	0.58	7.28	0.54	5.69	0.49	6.79	0.45	5.07
PR${}^{\left(\beta \;=\;1\right)}$	0.17	3.51	0.17	3.48	0.14	2.89	0.14	2.88	0.14	2.88	0.14	2.86
PR${}^{\left(\beta \;=\;2.5\right)}$	0.26	3.77	0.25	3.74	0.46	5.36	0.45	5.30	0.38	4.79	0.37	4.73
HSCNN-R${}^{\left(\beta \;=\;1\right)}$	0.18	4.12	0.15	3.75	0.18	3.91	0.12	2.92	0.18	4.06	0.12	2.95
HSCNN-R${}^{\left(\beta \;=\;10\right)}$	0.31	5.41	0.26	4.95	0.53	7.67	0.43	7.12	0.44	7.03	0.35	6.29
	$\Delta E_{00}$ **(Color Error)**											
	**SONY IMX135**				**NIKON D810**				**CANON 5DSR**			
	**Original**		**Physically Plausible**		**Original**		**Physically Plausible**		**Original**		**Physically Plausible**	
	**Mean**	**99.9 pt**	**Mean**	**99.9 pt**	**Mean**	**99.9 pt**	**Mean**	**99.9 pt**	**Mean**	**99.9 pt**	**Mean**	**99.9 pt**
LR	0.33	3.39	0.33	3.36	0.63	5.90	0.63	5.83	0.41	3.95	0.41	3.89
RPR	0.28	3.93	0.26	3.76	0.54	6.74	0.53	6.72	0.38	4.75	0.35	4.52
A+	0.27	4.93	0.24	4.37	0.49	8.33	0.45	7.86	0.34	5.88	0.30	5.29
RBFN${}^{\left(\beta \;=\;1\right)}$	0.43	10.75	0.23	4.75	0.56	13.01	0.39	8.12	0.47	11.55	0.26	5.46
RBFN${}^{\left(\beta \;=\;2.5\right)}$	0.36	4.92	0.30	3.33	0.71	6.48	0.66	5.26	0.47	5.22	0.40	3.50
PR${}^{\left(\beta \;=\;1\right)}$	0.23	4.34	0.23	4.33	0.42	8.17	0.43	8.15	0.27	5.38	0.27	5.37
PR${}^{\left(\beta \;=\;2.5\right)}$	0.24	3.52	0.23	3.53	0.51	6.11	0.50	6.16	0.33	3.94	0.32	3.97
HSCNN-R${}^{\left(\beta \;=\;1\right)}$	0.26	5.26	0.24	4.99	0.42	8.70	0.40	8.60	0.29	5.97	0.27	5.71
HSCNN-R${}^{\left(\beta \;=\;10\right)}$	0.35	5.75	0.28	5.10	0.62	9.36	0.56	9.06	0.43	6.40	0.36	5.97

## Supplementary Materials

The code of the methods introduced in this paper is available at https://github.com/EthanLinYitun/Physically_Plausible_Spectral_Reconstruction.
